# Evaluating the Potential of PSMA Targeting in CNS Tumors: Insights from Large-Scale Transcriptome Profiling

**DOI:** 10.3390/cancers17071239

**Published:** 2025-04-06

**Authors:** Adam Kraya, Komal Rathi, Run Jin, Varun Kesherwani, Adam C. Resnick, Phillip B. Storm, Ali Nabavizadeh

**Affiliations:** 1 Center for Data-Driven Discovery in Biomedicine (D3b), Children’s Hospital of Philadelphia, Philadelphia, PA 19104, USA; krayaa@chop.edu (A.K.);; 2 Division of Neurosurgery, Children’s Hospital of Philadelphia, Philadelphia, PA 19104, USA; 3 Department of Radiology, Perelman School of Medicine, University of Pennsylvania, Philadelphia, PA 19104, USA

**Keywords:** PSMA, transcriptomics, brain tumors

## Abstract

Background/Objectives: Prostate-specific membrane antigen (PSMA) is a well-established target in prostate cancer therapy that has shown potential as a theranostic target across non-central nervous system (CNS) and CNS tumor types. We aimed to investigate the pan-tissue expression pattern of the PSMA-encoding gene FOLH1 to assess whether transcriptome profiling can inform tumor diagnostic and theranostic probes. Methods: We assessed FOLH1 expression from the Open Pediatric Cancer Project (OpenPedCan, *n* = 2132 specimens), the Cancer Genome Atlas (TCGA, *n* = 10,411 specimens), and the Genotype Tissue Expression Project (GTEx, *n* = 17,382 specimens) in relation to published reports of PSMA radionuclide uptake in various tumors. Results: When comparing FOLH1 expression across tumor versus normal tissues, we found that non-CNS tumors exhibiting elevated expression of at least two-fold (FDR < 0.05) were reported to have significant PSMA radionuclide uptake in contrast to tumors with less than a two-fold elevation or with lower expression of FOLH1 relative to normal tissues. Notably, CNS tumors universally exhibited lower expression of FOLH1 relative to normal brain tissue, but we observed considerable variation in the expression of blood–tumor barrier (BTB) components associated with reports of BTB integrity and uptake of PSMA radiotracers. Conclusions: Large-scale transcriptomics data may help guide the application of PSMA-based radionuclide therapies in non-CNS tumors, but care should be taken to account for BTB effects in CNS tumors when assessing the potential for radionuclide success. This study demonstrates that FOLH1 showed a lack of tumor-specific expression for both adult and pediatric CNS tumors when compared to normal brain tissue, suggesting that PSMA is not a desirable target in brain tumors.

## Simple Summary

Prostate-specific membrane antigen (PSMA) is widely used in prostate cancer imaging and therapy, and there is growing interest in its potential applications across other tumor types. This study systematically analyzed large-scale gene expression datasets to assess whether FOLH1, the gene encoding PSMA, is differentially expressed in various cancers compared to normal tissues. The findings revealed that non-central nervous system (CNS) tumors with significantly elevated FOLH1 expression were associated with PSMA radiotracer uptake, suggesting they may be suitable candidates for PSMA-targeted therapies. However, CNS tumors exhibited lower FOLH1 expression relative to normal brain tissue, indicating a lack of tumor specificity. Moreover, variability in the integrity of the blood–tumor barrier may influence PSMA radiotracer uptake in brain tumors. These results suggest that while PSMA-based strategies hold promise for non-CNS tumors, their application in brain tumors may be limited due to biological constraints.

## 1. Introduction

Prostate-specific membrane antigen (PSMA) is a type II transmembrane glycoprotein that is expressed in various tissues [1]. Since the initial discovery of PSMA as a potential target for prostate cancer in the late 1980s [2], PSMA-targeting agents have been extensively studied for imaging and treatment of prostate cancer, which culminated in FDA approval [177Lu]Lu-PSMA-617 in March 2022 for treatment of adult patients with PSMA-positive metastatic castration-resistant prostate cancer (mCRPC) who have received previous chemo-therapy and are not responsive to hormone deprivation [3].

In recent years, there has been significant interest studying PSMA as an imaging biomarker and theranostic agent in non-prostate cancers [4,5,6,7,8,9,10,11,12,13,14,15,16]. Significant uptake of PSMA radiotracers has been observed across uterine, stomach, lung squamous cell carcinoma, ovarian, and thyroid cancers as well as within the renal clear cell carcinoma subtype of kidney cancer [5,7,8,9,11,15]. However, PSMA imaging and theranostics have failed in other cancer types such as lung adenocarcinomas, cervical squamous cell carcinomas, colorectal adenocarcinoma, as well as renal papillary cell carcinoma and kidney chromophobe subtypes [4,5,11,15,16]. PSMA theranostic approaches in CNS tumors have also garnered attention based on multiple studies that showed high PSMA expression in blood vessels of gliomas [17,18,19]. Despite the initial promising evidence from small case series of [18F]DCFPyL and 68Ga-PSMA PET imaging in high-grade gliomas and a dosimetry study in the treatment of a GBM patient with [177Lu]Lu-PSMA-617 [20,21,22], a recent study investigated [177Lu]Lu-PSMA therapy in high-grade glioma (HGG) with a focus on intratherapeutic dosimetry and demonstrated that only a minority of HGG patients had significant uptake on [68Ga]Ga-PSMA PET/MRI to be eligible for treatment with [177Lu]Lu-PSMA and among the treated patients, the achieved tumor dose was too low for a sufficient therapeutic effect [23]. In addition, in a recent study of 20 patients with HGG, only 3 patients demonstrated significant 68Ga-PSMA PET uptake to qualify for [177Lu]Lu-PSMA therapy [24]. Previous studies using immunohistochemistry demonstrated that astrocytes in various parts of normal brain express PSMA [1]. To our knowledge, there have yet to be investigations conducted to ascertain an underlying molecular etiology that may explain the variability in uptake and dosimetry for PSMA theranostics.

Given that the level of PSMA protein expression has been previously observed to associate with uptake and response to radiolabeled PSMA-targeting ligands both in vitro and in prostate cancer trials [25,26,27], we hypothesized that there exists a positive association between the RNA expression levels of PSMA and responses to radioligand interventions across tumor types. We further hypothesized that the expression of FOLH1 in tumor relative to normal tissue may differentiate between tumor histologies that have historically responded to PSMA radioligand therapies or demonstrated significant uptake on diagnostic PSMA PET imaging versus those that did not. In this study, we performed a comprehensive bioinformatics analysis of publicly available databases to get a better understanding of FOLH1/PSMA expression in adult and pediatric CNS and non-CNS tumors in comparison to normal tissues. Our goal was to determine the potential for large-scale expression data derived from multi-institutional consortia to predict PSMA radiotracer uptake in adult and pediatric tumors, assess the potential for predicting uptake of PSMA radiotracers using FOLH1 gene expression in normal tissues, and discern whether the expression of blood–brain barrier and blood–tumor barrier components may give insights into differential uptake of PSMA radiotracers in CNS tumors.

## 2. Materials and Methods

We obtained the transcripts-per-million (TPM) expression of FOLH1 (PSMA encoding gene) across samples from three resources: 57 pediatric solid tissue tumor types from the latest version (v12) of OpenPedCan-analysis derived from the Open Pediatric Brain Tumor (Open-PBTA) project [28] (*n* = 2132 specimens), 34 adult solid tissue tumor types from the Cancer Genome Atlas (TCGA) [29] (*n* = 10,411 specimens), and 31 normal tissue types from the Genotype Tissue Expression Project (GTEx) [30] (*n* = 17,382 specimens). All samples were processed and harmonized through a standardized Kids First pipeline using STAR alignment followed by RNA-seq by expectation-maximization (RSEM) quantification to generate TPM and count expression data [28,31].

To gain a deeper understanding of the potential for PSMA-targeting theranostics across tumor types, we evaluated FOLH1 expression patterns across all cancer cohorts relative to their corresponding matched normal tissue as well as in comparison to dose-limiting tissues that were available on the GTEx database (kidney, liver, minor salivary gland, small intestine, and spleen). To statistically compare gene expression of FOLH1 across tumor and normal tissues, we performed differential gene expression analysis across all protein-coding genes (Gencode v39) using the software package Differential Expression Analysis for Sequence Count Data (DESeq2) [32], leveraging expected counts from RSEM as input and correcting for latent batch effects using surrogate variables within the statistical design. We included the histology of the tumor and the GTEx tissue group for all tumor-normal pairwise comparisons. We evaluated log fold change, Benjamini–Hochberg adjusted p-value, and the directionality of the expression change for FOLH1 across all tumor-normal comparisons. To investigate the potential for expression data to predict radiotracer uptake and dosimetry, we compiled previously published mean or median (depending on availability) PSMA radiotracer uptake measurements (mGy/MBq) in normal tissue [33,34] and calculated the Spearman correlation with mean FOLH1 expression in the matching normal GTEx tissue type.

To infer brain–tumor barrier (BTB) and blood–brain barrier (BBB) disruption, we compared the expression of genes encoding for known junctional and transporter proteins that comprise the neuro-vascular unit (NVU) [35] relative to normal brain expression by differential gene expression analysis. Disruption in the BTB or BBB was defined by a tumor cohort exhibiting a differential expression in more than one gene in the expected direction known to compromise BTB/BBB function.

## 3. Results

**Figure 1:**
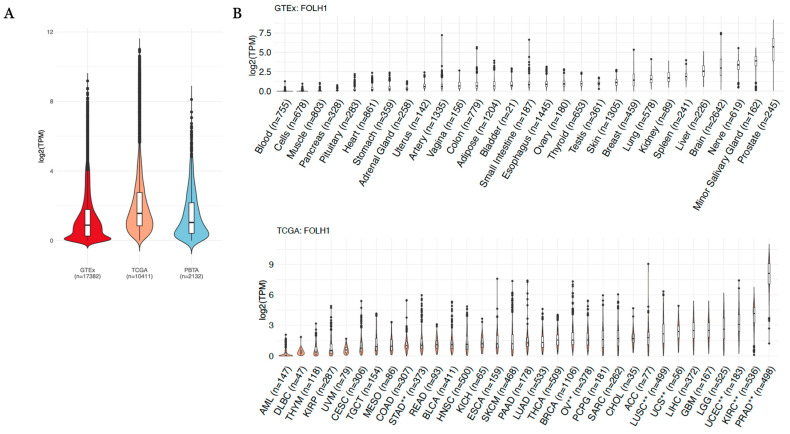
(**A**) Expression (log2(TPM)) of FOLH1 across pan-GTEx, pan-TCGA, and pan-PBTA cohorts. (**B**) Expression (log2(TPM)) of FOLH1 across pan-GTEx and pan-TCGA cohorts broken down by tissue or tumor type. ** Cancer type with previously reported favorable response to PSMA radioligand therapy or significant uptake on diagnostic PSMA PET imaging.

Comparing the expression of FOLH1, we observed a less than two-fold difference in the median expression of FOLH1 across 31 GTEx normal tissues (median log2 TPM = 0.886) compared to 57 PBTA pediatric solid tumor types (median log2 TPM = 1.04) and 34 TCGA adult solid tumor types (median log2 TPM = 1.56) (Figure 1A). When broken down by tissue type across TCGA and GTEx and filtered for tissues or histologies with greater than 20 samples (prostate adenocarcinoma (PRAD), stomach adenocarcinoma (STAD), uterine carcinosarcoma and uterine corpus endometrial carcinoma (UCS, UCEC), kidney renal clear cell carcinoma (KIRC), lung squamous cell carcinoma (LUSC), ovarian serous cystadenocarcinoma (OV), and thyroid carcinoma (THCA) cancers) demonstrate at least a fold change of two or greater by differential gene expression analysis and when comparing median TPM expression in the tumor relative to corresponding normal tissue (Figure 1B, Table 1). Notably, PRAD from TCGA illustrated a median log2 TPM of 8.13 relative to a median log2 TPM of 5.70 in normal GTEx prostate tissue. Further, all tumor types along with their corresponding normal tissue, with the exception of PRAD, exhibited median expression values below 5 TPM on a log2 scale. Kidney cancer subtypes varied considerably in terms of their FOLH1 expression relative to normal kidney tissue, with papillary and chromophobe carcinomas (KIRP and KICH, respectively) exhibiting underexpression and kidney clear cell carcinoma (KIRC) showing overexpression (Table 1).

**Figure 2:**
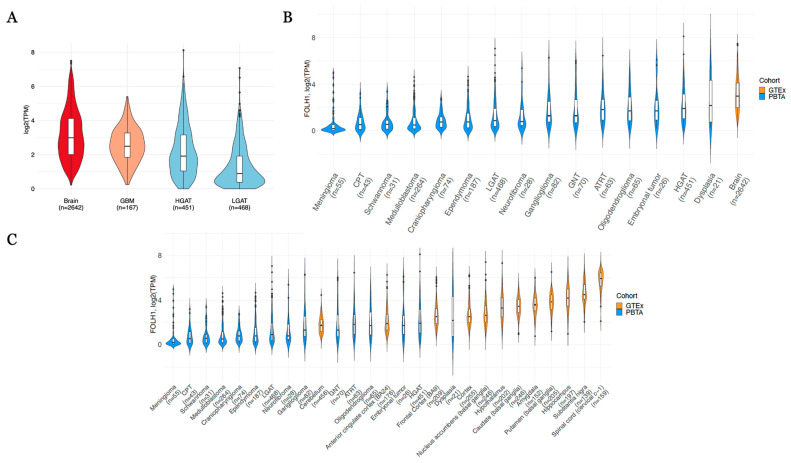
(**A**) Expression (log2(TPM)) of FOLH1 comparing normal Brain (GTEx), TCGA glioblastoma (GBM), pediatric high-grade gliomas (HGG, PBTA), and pediatric low-grade gliomas (LGG, PBTA). (**B**) Expression (log2(TPM)) of FOLH1 comparing pan-GTEx to histologies of the PBTA and (**C**) when comparing pan-GTEx broken down by tissue type to tumor histologies of the PBTA.

When comparing only GTEx brain tissue to TCGA GBM (median log2 TPM = 2.49), high-grade astrocytomas (HGATs) of the PBTA, and low-grade astrocytomas (LGATs) of the PBTA, we found higher expression in normal brain tissues (median log2 TPM = 2.99) compared to tumor tissues (Figure 2A). When extending to all 15 pediatric brain tumor types (n ≥ 20), we observed that all pediatric brain tumors exhibit generally low FOLH1 expression levels, with a median log2 TPM of less than five (Figure 2B). Figure 2C further illustrates that the majority of normal brain regions express FOLH1 at levels higher than brain tumors, including pediatric LGAT and HGAT cancers. This was confirmed statistically by differential expression analysis, whereby we observed higher expression in GTEx normal brain tissue when comparing to both adult low-grade gliomas (LGG) and GBM (Table 1). Further, all pediatric central nervous system tumors of the PBTA exhibited significantly lower expression of FOLH1 relative to GTEx brain tumor tissue (Table 2), underscoring the lack of tumor specificity for this target in brain tumors.

**Figure 3:**
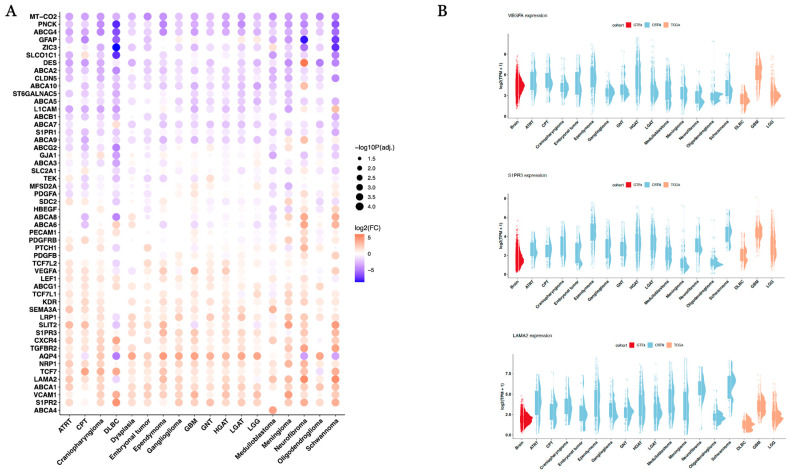
(**A**) Differential gene expression (TCGA/PBTA versus GTEx normal brain) of blood tumor barrier proteins. (**B**) Raincloud plots of VEGFA, S1PR3, and LAMA2 expression (log2(TPM)) across adult and pediatric brain tumors.

However, as adult GBM’s have shown high tumor-to-background ratios for PSMA radionuclides despite our observation of higher expression in normal brain tissue [17,18,19,20,21,36], we chose to investigate the expression of junctional and transporter proteins known to comprise the neuro-vascular unit (NVU) and regulate the permeability of the blood–brain and blood–tumor barriers (BBB and BTB) [35]. We investigated the expression across all adult and pediatric brain tumor types, focusing in particular on GBM and pediatric HGAT as well as pediatric LGAT and adult low-grade gliomas (LGGs) given the observations of superior radionuclide uptake and higher BTB permeability in the former relative to the latter [22,37]. Through differential gene expression analysis of each brain tumor type relative to GTEx normal brain tissue, we found that GBM (logFC: 3.06, *p* < 1 × 10^−300^) and pediatric HGAT (logFC: 2.17, *p* < 1 × 10^−300^) expressed VEGFA, a known regulator of BTB permeability in GBM [38], at significantly higher levels than normal brain tissue, but adult LGG showed a substantially lower degree of overexpression relative to high-grade tumors (logFC: 0.46, *p* = 9.75 × 10^−17^), and pediatric LGAT (logFC: −0.06, *p* = 0.19) did not demonstrate differential expression relative to normal brain (Figure 3A,B). S1PR3, which encodes for the sphingosine 1-phosphate receptor 3, and LAMA2, which encodes for the BTB junctional protein laminin subunit alpha 2, both known biomarkers of a permeable BTB [31], were overexpressed in adult GBM relative to normal brain tissue, while adult LGG showed underexpression of LAMA2 and a lower degree of S1PR3 overexpression relative to adult GBM (Table 3, Figure 3B). In pediatric gliomas, while LGAT was found to overexpress S1PR3 to a slightly greater degree than HGAT, LGAT had comparatively lower overexpression of LAMA2 than that of HGAT (Table 3, Figure 3B). Collectively, with the minor exception of S1PR3 in pediatric tumors, BTB expression patterns suggested a more permeable phenotype in high-grade relative to low-grade gliomas. Across all pediatric and adult brain tumors, we found a subset of ATP-binding cassette (ABC) transporters, which play an important role in mediating efflux of xenobiotics, thereby regulating the permeability of the NVU [35], to be mostly underexpressed relative to normal brain tissue (ABCG4, ABCA2, ABCA10, ABCA5, ABCB1, ABCA7, ABCA9, ABCG2, ABCA3), while ABCA8 and ABCA6 showed variable patterns of expression across the set of tumor histologies (Figure 3A). Notably, ABCG1, ABCA1, and ABCA4 were universally overexpressed relative to normal brain tissue, indicating these genes may play a particularly important role in the efflux of xenobiotics relative to the remaining transporters (Figure 3A).

**Figure 4:**
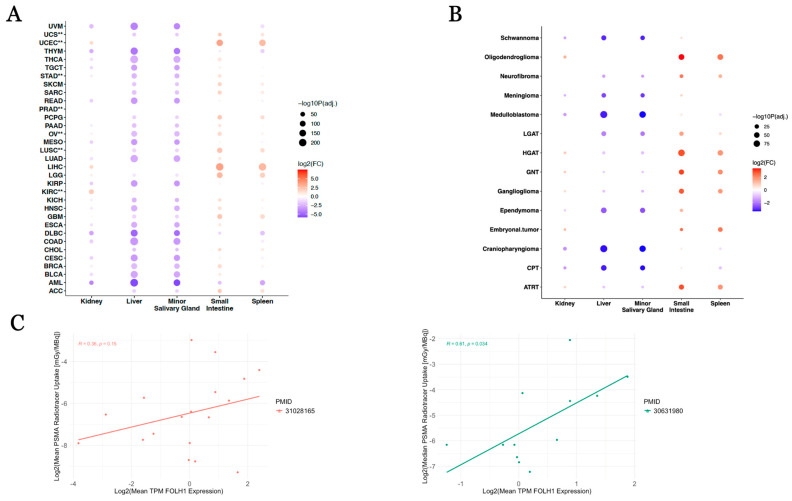
(**A**) Differential FOLH1 expression across adult TCGA tumors versus dose-limiting normal tissues from GTEx. (**B**) Differential FOLH1 expression across pediatric PBTA tumors versus dose-limiting normal tissues from GTEx. (**C**) Association of FOLH1 expression with reported PSMA radiotracer uptake from two independent dosimetry studies. ** Cancer type with previously reported favorable response to PSMA radioligand therapy or significant uptake on diagnostic PSMA PET imaging.

We then comparatively evaluated the expression of FOLH1 across all tumor types relative to dose-limiting tissues based on the availability of data in the GTEx database, which included the kidney, liver, minor salivary gland, small intestine, and spleen. Among uterine tumors that showed elevated expression relative to their corresponding normal tissues (UCEC—uterine corpus endometrial carcinoma, UCS—uterine carcinosarcoma), UCS showed lower expression of FOLH1 relative to the liver (log2FC = −1.74, *p* = 8.42 × 10^−26^) and minor salivary gland (log2FC = −1.75, *p* = 3.64 × 10^−19^), as opposed to UCEC which showed similar levels of expression relative to liver (log2FC = −0.18, *p* = 0.14) and minor salivary glands (log2FC = −0.18, *p* = 0.24) and higher expression relative to kidney (log2FC = 1.59, *p* = 8.25 × 10^−20^), small intestine (log2FC = 3.58, *p* = 2.13 × 10^−134^), and spleen (log2FC = 2.67, *p* = 5.58 × 10^−137^) (Figure 4A, Supplementary Table S1); notably, UCS also exhibited elevated expression of FOLH1 relative to small intestine (log2FC = 2.02, *p* = 5.72 × 10^−24^) and spleen (log2FC = 1.08, *p* = 1.00 × 10^−19^) but similar levels to kidney (log2FC = 0.03, *p* = 0.86). When comparing kidney cancers, KIRC illustrated higher FOLH1 expression relative to normal kidney (log2FC = 2.03, *p* = 2.69 × 10^−56^), liver (log2FC = 0.33, *p* = 1.73 × 10^−4^), salivary gland (log2FC = 0.24, *p* = 0.019), small intestine (log2FC = 3.99, *p* < 1 × 10^−300^), and spleen (log2FC = 3.13, *p* < 1 × 10^−300^). KIRP (log2FC = −1.70, *p* = 2.25 × 10^−17^) and KICH (log2FC = −0.65, *p* = 3.57 × 10^−4^) showed lower expression of FOLH1 relative to normal kidney. Notably, KIRP only showed overexpression relative to small intestine (log2FC = 0.39, *p* = 0.011, while KICH showed overexpression relative to small intestine (log2FC = 1.42, *p* = 2.79 × 10^−14^) and spleen only (log2FC = 0.51, *p* = 3.19 × 10^−4^; Supplementary Table S1), consistent with past studies that observed poorer uptake and tumor to background ratio in papillary and chromophobe kidney carcinomas relative to kidney clear cell carcinoma [4,5]. With the exception of prostate adenocarcinoma (PRAD), liver hepatocellular carcinoma (LIHC), and the aforementioned uterine and kidney cancer types, all remaining cancer types showed lower expression in the salivary gland and liver, and among solid tumors, uveal melanoma (UVM), rectal adenocarcinoma (READ), and thymomas (THYM) had lower expression of FOLH1 in the tumor relative to the spleen (Figure 4A, Supplementary Table S1). While stomach adenocarcinoma showed far higher FOLH1 expression than normal stomach tissue (Table 1), expression was lower relative to kidney (log2FC = −0.71, *p* = 1.33 × 10^−4^), liver (log2FC = −2.38, *p* = 1.79 × 10^−81^), and salivary gland (log2FC = −2.47, *p* = 2.39 × 10^−63^; Supplementary Table S1). Among pediatric CNS tumors, all illustrated lower expression of FOLH1 in at least two dose-limiting tissues with the exception of oligodendrogliomas, whose FOLH1 expression was either similar to or higher than that of the dose-limiting tissues of interest (Figure 4B, Supplementary Table S2). To assess the potential of our method to predict dosimetry, we evaluated the correlation of mean normal FOLH1 tissue expression (TPM) relative to previously published measurements of PSMA radiotracer uptake in normal tissues [33,34]. From an evaluation of low-risk prostate cancer patients by Sandgren et al., we found a significantly positive correlation between mean FOLH1 expression and the reported median PSMA radiotracer uptake (Figure 4C (green); *p* = 0.034, R = 0.61) and a non-significant positive correlation between mean FOLH1 expression and mean PSMA uptake in the study published by Piron et al. (Figure 4C (red); *p* = 0.15, R = 0.36).

## 4. Discussion

We aimed to determine whether comparisons of FOLH1 expression derived from large-scale transcriptomic profiling of tumor cohorts and of related normal tissue could determine tumor types for which PSMA-targeting theranostics may have utility. Our study was motivated in part by the observation that diverse therapies, including androgen deprivation, enzalutamide, and rapamycin, can increase the expression of PSMA protein expression, increase uptake of PSMA radioligands, and lead to improved anti-tumor responses in patients [25,26,27]. We, therefore, aimed to evaluate whether histology-specific expression of FOLH1 may show patterns suggestive of tumor specificity for tumor types known to respond well to PSMA radioligand interventions or show significant uptake on diagnostic PSMA PET imaging. We did so by comparing expression across tumor and normal tissue, particularly focusing on differential tumor uptake and tumor-to-background ratio. We found that prostate adenocarcinoma had among the highest differences in expression of FOLH1 relative to its matched normal tissue, consistent with past investigations involving the use of PSMA-targeting theranostics and their successful implementation within the disease [6]. Even more compelling was the general observation that tumors with a FOLH1 expression fold change of two or greater relative to their matched normal tissue were previously observed to have favorable uptake and tumor-to-background ratio, including bladder (BLCA), thyroid (THCA), uterine (UCS, UCEC), ovarian (OV), and stomach carcinomas (STAD) [4,7,8,9,10,11,12,13,14,15,16]. By contrast, lung adenocarcinomas (LUAD), colorectal adenocarcinomas (COAD), and breast carcinomas (BRCA) overexpressed FOLH1 at a fold change of less than two, and these cancers have previously shown relatively low uptake and tumor-to-background ratios [15,16]. Our observations with kidney cancer subtypes further illustrated the specificity of our approach, as papillary and chromophobe carcinomas showed underexpression of FOLH1 relative to normal kidney tissue, consistent with studies that showed poor uptake and tumor-to-background ratio in these tumor types [4,15]. By contrast, kidney clear cell carcinomas showed a fold change greater than four relative to normal kidney, and prior studies have shown successful implementation of PSMA radioligands for these tumors [5]. Taken together, these results demonstrate the potential positive and negative predictive value of large-scale expression data with respect to target selection for radioligand interventions in non-CNS tumors. Furthermore, we illustrated a positive correlation between the expression of FOLH1 in healthy tissue and the uptake of PSMA radionuclide in corresponding tissues. This highlights the potential ability to inform on dosimetry based on gene expression with the caveat that transcriptomic analysis can only inform on specific binding and absorbed dose per organ also depends on various other factors such as post-transcriptional modifications, tumor microenvironment (blood flow, nonspecific binding), and proximal tubule reabsorption [33,35].

While there has been much interest in the use of PSMA radiotracers in gliomas [15,18,19,20,22,36], our analysis demonstrated that FOLH1 showed a lack of tumor-specific expression for both adult and pediatric CNS tumors when compared to normal brain tissue, suggesting that PSMA is not a desirable target in this disease type. While prior work has shown that GBMs express relatively high levels of PSMA in the tumor neovasculature [17,18], these studies did not specify whether PSMA was observed on the apical or basal side of the vascular wall. Further, these studies were limited by a low sample size and a high degree of variability in the vascular expression of PSMA, a fact acknowledged by the authors. In a recent study of [18F]rhPSMA-7.3 in GBM-bearing mice using PET pharmacokinetic modeling, the time activity curves of the tumors demonstrated an initial uptake, followed by a wash-out, and K3 and k4 did not show significant differences, indicating no radiotracer trapping [36]. Further complicating expression analyses of CNS tumors in relation to radionuclide therapy potential is the fact that high-grade gliomas have shown high tumor-to-background ratios in past studies despite our expression data indicating higher expression in normal tissue relative to tumor. This compelled us to explore the expression of BTB components as potential predictive biomarkers of brain tumor radionuclide uptake. Our investigation indicated that well-established markers of BTB permeability, namely VEGFA, S1PR3, and LAMA2, showed expression patterns across adult and pediatric high- and low-grade gliomas that suggested greater BTB permeability in high grades, consistent with past comparative BTB assessments in adult HGG and LGG [37]. Our observation is also consistent with a past PSMA radiotracer study demonstrating that GBM achieved superior uptake relative to adult LGGs [22]. Taken together, these results suggest the expression of BTB genes may carry predictive value when considering the potential for radionuclide therapies in tandem with target expression.

While our study highlights that bulk transcriptome expression analysis across large cancer cohorts has both positive and negative predictive value, PSMA assessments in brain tumors may benefit further from single-cell sequencing analyses, a data modality that was not available for this study. A recent study in breast cancer highlighted that specific PSMA isoforms may be selectively and highly expressed among breast cancer stem cells (BCSCs), which represent a subpopulation of tumor cells that promote cancer recurrence and progression due to their multi-potency and self-renewing capacity [39] Notably, BCSCs showed greater uptake of radiolabeled PSMA, consistent with higher expression relative to the remaining non-stem tumor cell populations. This observation suggests that single-cell sequencing analyses may help to identify a subset of GBMs harboring elevated PSMA expression in the GBM stem cell subpopulation, which are known to play a pivotal role in radio- and chemo-therapy resistance [40]. Presumably, a precision medicine approach may entail the use of PSMA-targeting radionuclides in a subgroup of GBM patient tumors showing elevated expression of PSMA in resistant stem cells. Another limitation of our study had to do with the lack of availability of matched patient RNA-seq and PSMA PET imaging data. Due to the lack of matched data, we were unable to derive a true predictive model between FOLH1 expression and PSMA radiotracer uptake, and we were limited to correlative assessments driven by analysis of publicly available RNA-seq data and curation of previously published PSMA uptake data. This further limited us in our assessments of BBB/BTB effects, as we are unable to confirm that RNA-seq-based measures of BBB/BTB disruption definitively correlate with uptake of PSMA radiotracers. Lastly, we were unable to evaluate correlations between RNA-based FOLH1 expression and proteomics-based PSMA expression due to the fact that PSMA was not profiled as part of the proteomics characterization efforts from the CBTN or GTEx consortium.

## 5. Conclusions

This study emphasizes the need and utility for cross-referencing large-scale molecular data derived from sequencing consortia to guide the selection of theranostic targets for preclinical and clinical evaluation as part of an imaging-genomic research paradigm. An integrative imaging-genomic approach can predict the potential for success when investigating novel theranostic targets, thereby potentially accelerating the development of imaging probes and radionuclide therapies for the clinical benefit of cancer patients.

## Figures and Tables

**Table 1:** Results of differential expression analysis (DESeq2) across adult TCGA cancers versus matched normal tissues from GTEx. ** Cancer type with previously reported favorable response to PSMA radioligand therapy.

*FOLH1* Comparison	Log2(Fold Change)	Direction vs. Normal Tissue	Adjusted *p*-Value
GBM_vs._Brain	−1.734	Down	5.84 × 10^−43^
KIRP_vs._Kidney	−1.699	Down	2.25 × 10^−17^
ACC_vs._Minor.Salivary.Gland	−1.551	Down	8.74 × 10^−17^
LGG_vs._Brain	−1.290	Down	1.26 × 10^−64^
KICH_vs._Kidney	−0.645	Down	3.57 × 10^−4^
LUAD_vs._Lung	0.534	Up	9.12 × 10^−17^
BRCA_vs._Breast	0.742	Up	3.80 × 10^−20^
READ_vs._Colon	0.853	Up	5.16 × 10^−9^
COAD_vs._Colon	0.958	Up	1.42 × 10^−29^
BLCA_vs._Bladder	1.052	Up	6.99 × 10^−4^
ESCA_vs._Esophagus	1.240	Up	7.33 × 10^−47^
CESC_vs._Vagina	1.507	Up	1.24 × 10^−25^
SKCM_vs._Skin	1.885	Up	2.44 × 10^−165^
THCA_vs._Thyroid	1.896	Up	4.10 × 10^−260^
LUSC **_vs._Lung	2.009	Up	6.41 × 10^−153^
KIRC **_vs._Kidney	2.033	Up	2.69 × 10^−56^
OV **_vs._Ovary	2.119	Up	5.11 × 10^−82^
PRAD **_vs._Prostate	2.743	Up	5.65 × 10^−134^
STAD **_vs._Stomach	2.829	Up	1.66 × 10^−130^
UCS **_vs._Uterus	3.530	Up	9.72 × 10^−92^
UCEC **_vs._Uterus	5.103	Up	7.75 × 10^−269^

**Table 2:** Results of differential expression analysis (DESeq2) across pediatric PBTA cancers versus brain tissue from GTEx.

*FOLH1* Comparison	Log2(Fold Change)	Direction vs. Normal Tissue	Adjusted *p*-Value
Craniopharyngioma_vs._Brain	−3.570	Down	2.47 × 10^−79^
CPT_vs._Brain	−3.549	Down	1.17 × 10^−46^
Schwannoma_vs._Brain	−3.527	Down	9.30 × 10^−28^
Medulloblastoma_vs._Brain	−3.504	Down	9.08 × 10^−239^
Meningioma_vs._Brain	−2.823	Down	7.94 × 10^−33^
Ependymoma_vs._Brain	−2.715	Down	1.03 × 10^−103^
LGAT_vs._Brain	−2.143	Down	1.01 × 10^−143^
Neurofibroma_vs._Brain	−1.761	Down	5.61 × 10^−8^
Embryonal tumor_vs._Brain	−1.751	Down	3.93 × 10^−6^
Ganglioglioma_vs._Brain	−1.398	Down	4.43 × 10^−14^
HGAT_vs._Brain	−1.340	Down	1.40 × 10^−47^
ATRT_vs._Brain	−1.096	Down	1.05 × 10^−7^
GNT_vs._Brain	−1.080	Down	2.18 × 10^−7^
Oligodendroglioma_vs._Brain	−0.551	Down	7.94 × 10^−3^

**Table 3:** Results of differential expression analysis (DESeq2) across adult TCGA and pediatric PBTA HGG and LGG cancers versus brain tissue from GTEx for select BTB genes.

Comparison	Gene	Log2(Fold Change)	Direction Versus Normal Brain Tissue	Adjusted *p*-Value
GBM_vs._Brain	LAMA2	0.93	Up	1.52 × 10^−47^
GBM_vs._Brain	S1PR3	2.51	Up	2.64 × 10^−171^
GBM_vs._Brain	VEGFA	3.06	Up	<1 × 10^−300^
HGAT_vs._Brain	LAMA2	2.06	Up	<1 × 10^−300^
HGAT_vs._Brain	S1PR3	1.68	Up	8.94 × 10^−184^
HGAT_vs._Brain	VEGFA	2.17	Up	<1 × 10^−300^
LGAT_vs._Brain	LAMA2	1.45	Up	2.45 × 10^−216^
LGAT_vs._Brain	S1PR3	1.88	Up	5.32 × 10^−251^
LGAT_vs._Brain	VEGFA	0.42	Up	9.75 × 10^−17^
LGG_vs._Brain	LAMA2	−0.32	Down	2.79 × 10^−15^
LGG_vs._Brain	S1PR3	1.38	Up	1.05 × 10^−136^
LGG_vs._Brain	VEGFA	−0.06	Down	0.19

## Data Availability

De-identified transcriptomic source data as well as clinical data, including patient baseline characteristics, molecular subtypes, and outcomes, analyzed for this study are available in dbGaP study phs002517.v2.p2 (https://www.ncbi.nlm.nih.gov/projects/gap/cgi-bin/study.cgi?study_id=phs002517.v2.p2, accessed on 4 September 2024) as well as through the Kids First Data Resource Portal (https://portal.kidsfirstdrc.org/studies, accessed on 4 September 2024) under study id phs002517. The processed data are available at https://github.com/d3b-center/OpenPedCan-analysis, accessed on 4 September 2024.

## Supplementary Materials

The following supporting information can be downloaded at: https://www.mdpi.com/article/10.3390/cancers17071239/s1, Table S1: Results of Differential Expression Analysis (DESeq2) Across Adult TCGA Cancers Versus Matched Normal Tissues from GTEx; Table S2: Results of Differential Expression Analysis (DESeq2) Across Pediatric PBTA Cancers Versus Brain Tissue from GTEx; Table S3: Differential Expression Analysis (DESeq2) Across Adult TCGA and Pediatric PBTA HGG and LGG Cancers versus Brain Tissue from GTEx for select BTB genes; Table S4: Differential Expression of FOLH1 in TCGA Tumors Relative to Dose-Limiting Normal Tissues; Table S5: Differential Expression of FOLH1 in PBTA Tumors Relative to Dose-Limiting Normal Tissues.

## Abbreviations

The following abbreviations are used in this manuscript: AML = acute myeloid leukemia, DLBC = lymphoid neoplasm diffuse large B-cell lymphoma, THYM = thymoma, KIRP = kidney renal papillary cell carcinoma, UVM = uveal melanoma, CESC = cervical squamous cell carcinoma and endocervical adenocarcinoma, TGCT = testicular germ cell tumors, MESO = mesothelioma, COAD = colon adenocarcinoma, STAD = stomach adenocarcinoma, READ = rectum adenocarcinoma, BLCA = bladder urothelial carcinoma, HNSC = head and neck squamous cell carcinoma, KICH = kidney chromophobe, ESCA = esophageal carcinoma, SKCM = skin cutaneous melanoma, PAAD = pancreatic adenocarcinoma, LUAD = lung adenocarcinoma, THCA = thyroid carcinoma, BRCA = breast invasive carcinoma, OV = ovarian serous cystadenocarcinoma, PCPG = pheochromocytoma and paraganglioma, SARC = sarcoma, CHOL = cholangiocarcinoma, ACC = adrenocortical carcinoma, LUSC = lung squamous cell carcinoma, LIHC = liver hepatocellular carcinoma, GBM = glioblastoma multiforme, LGG = low-grade astrocytoma, UCEC = uterine corpus endometrial carcinoma, UCS = uterine carcinosarcoma, KIRC = kidney renal clear cell carcinoma, PRAD = prostate adenocarcinoma, ATRT: atypical teratoid rhabdoid tumor, GNT: neuronal and mixed neuronal-glial tumor, CPT: choroid plexus tumor, HGAT = high-grade astrocytoma.
